# Sarcopenia and Risk of Pancreatic Fistula after Pancreatic Surgery: A Systematic Review

**DOI:** 10.3390/jcm11144144

**Published:** 2022-07-16

**Authors:** Teresa Perra, Giovanni Sotgiu, Alberto Porcu

**Affiliations:** Department of Medical, Surgical and Experimental Sciences, University of Sassari, Viale San Pietro 43, 07100 Sassari, Italy; gsotgiu@uniss.it (G.S.); alberto@uniss.it (A.P.)

**Keywords:** pancreatic surgery, sarcopenia, postoperative pancreatic fistula, CT measurements, pancreatoduodenectomy, distal pancreatectomy, Whipple, skeletal muscle

## Abstract

Postoperative pancreatic fistula (POPF) is one of the most critical complications after pancreatic surgery. The relationship between sarcopenia and outcomes following this type of surgery is debated. The aim of this review was to assess the impact of sarcopenia on the risk of POPF. A literature search was performed using the PubMed database and the reference lists of relevant articles to identify papers about the impact of sarcopenia on POPF in pancreatic surgery. Twenty-one studies published between 2016 and 2021 with a total of 4068 patients were included. Some studies observed a significant difference in the incidence of POPF between the sarcopenic and non-sarcopenic patients undergoing pancreatoduodenectomy. Interestingly, there was a trend of a lower POPF rate in sarcopenic patients than in non-sarcopenic patients. Only one study included patients undergoing distal pancreatectomy specifically. The role of sarcopenia in surgical outcomes is still unclear. A combination of objective CT measurements could be used to predict POPF. It could be assessed by routine preoperative staging CT and could improve preoperative risk stratification in patients undergoing pancreatic surgery.

## 1. Introduction

Pancreatic surgery is technically complex and associated with significant postoperative morbidity, mortality, and prolonged hospitalisation. In recent decades, although survival after pancreatic surgery has improved due to recent advancements in perioperative management and operative technique, many patients still develop complications.

Pancreatoduodenectomy is the gold standard in the treatment of pancreatic, periampullary, and distal bile duct malignancies and should only be performed in centres with high expertise in this type of surgery. Postoperative pancreatic fistula (POPF) is one of the most common and relevant complications following this procedure.

Many possible risk factors of POPF have been identified, such as male gender, higher body mass index, prior history of cholangitis, cardiovascular disease, benign rather than malignant indication, extra-pancreatic tumour location, blood loss, soft parenchymal texture, narrow pancreatic duct width (<3 mm), absence of intraoperative blood transfusion, and higher fluid amylase on postoperative day 1 [1]. 

The evaluation of the nutritional status of patients undergoing pancreatic surgery has been receiving increasing attention, especially in recent years, and according to a position paper of the International Study Group on Pancreatic Surgery (ISGPS), the measurement of nutritional status should be part of the routine preoperative assessment, as malnutrition is a known risk factor for surgery-related complications. The group also suggests considering, in addition to the patient’s weight loss and body mass index, the measurement of sarcopenia and sarcopenic obesity [2,3].

Sarcopenia seems to be associated with poorer survival, higher postoperative morbidity, and mortality in patients undergoing pancreatic surgery. It can be assessed by the routine preoperative staging CT, but its role in surgical outcomes is still unclear. In particular, its role in the occurrence of POPF is debated.

There is increasing evidence that sarcopenia should be considered in the preoperative risk assessment and treatment decision making in patients undergoing pancreatic surgery [4,5].

Predicting POPF using a combination of objective preoperative CT measurements could be very useful. Body composition parameters could be evaluated routinely, easily, and at no additional cost.

The aim of our study was to assess the impact of sarcopenia on the risk of POPF after pancreatic surgery, and following pancreatoduodenectomy and distal pancreatectomy specifically.

## 2. Materials and Methods

### 2.1. Literature Search

**Figure 1:**
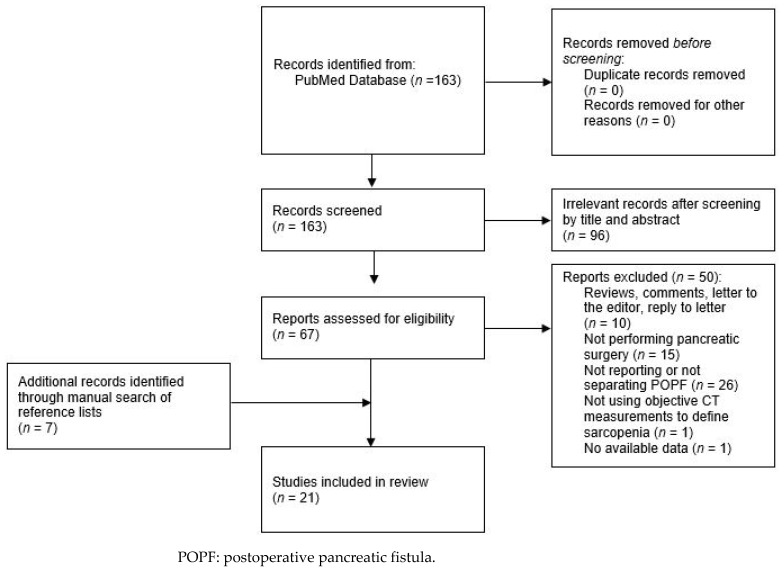
PRISMA flow diagram of the literature search strategy.

A literature search was performed using the PubMed database, until December 2021, by two independent investigators (T.P., A.P.). The following search terms were used: (“pancreaticoduodenectomy” OR “pancreatoduodenectomy” OR “Whipple” OR “distal pancreatectomy” OR “pancreatic surgery”) AND (“sarcopenia” OR “skeletal muscle” OR “body composition”). A manual search of the reference lists in relevant articles was also conducted to identify additional studies. No language restriction was applied. The literature search strategy is presented in Figure 1.

### 2.2. Inclusion and Exclusion Criteria

Studies were included if they compared POPF between sarcopenic and non-sarcopenic patients who underwent pancreatic resection and used preoperative objective CT measurements to define sarcopenia. Studies were excluded if they were animal studies, case reports, comments, or letters to the editor, or if they did not provide separate outcomes for sarcopenic and non-sarcopenic patients.

### 2.3. Data Extraction and Quality Assessment

Extracted data were recorded by the authors using a standardised template including the following: author, publication year, study design, indication for pancreatic resection, type of resection, sample size, sample population details, imaging technique, radiographic definition of sarcopenia, and cut-off values for sarcopenia. The number of POPFs in each study was also recorded.

The study quality was assessed using the Newcastle–Ottawa scale (NOS) for cohort studies [6].

### 2.4. Terminology and Definitions

Sarcopenia was radiologically defined as a low skeletal muscle mass diagnosed by the examination of a single axial cross-sectional image at the third lumbar vertebra level on preoperative CT. It was generally recorded as a measurement of the skeletal muscle index (SMI), but also as the total abdominal muscle area index (TAMAI), skeletal muscle area divided by the body surface area (SBI), muscle radiation attenuation (MRA), psoas muscle mass index (PMI), HUAC (Hounsfield unit average calculation) of the psoas muscles, or intramuscular adipose tissue content (IMAC).

POPF was defined according to the International Study Group for Pancreatic Fistula classification [7].

No meta-analysis was carried out based on the heterogeneity of the study variables and design.

## 3. Results

Twenty-one studies published between 2016 and 2021 with a total of 4068 patients were included. Seventeen studies were retrospective and four were prospective. The total number of patients diagnosed with sarcopenia was 1921, and that of non-sarcopenic patients was 2147.

Study characteristics are reported in Table 1.

The skeletal muscle index (SMI) at the third lumbar vertebra level on preoperative CT was the most common way of assessing sarcopenia, although the cut-offs varied among different studies. The different measurements and cut-offs used to define sarcopenia are reported in Table 2.

Some studies observed a statistically significant effect of sarcopenia, as shown in Table 3. The occurrence of POPF was found to be similar between sarcopenic and non-sarcopenic groups (see Table 4).

The indications for surgery and types of resection were different. Seventeen studies included patients undergoing pancreatoduodenectomy, such as Whipple’s procedure and pylorus-preserving pancreatoduodenectomy. Interestingly, there was a trend of a lower POPF rate in sarcopenic patients than in non-sarcopenic patients (see Table 5). POPF severity using the ISGPF classification is reported in Table 6.

Only one study included patients who underwent distal pancreatectomy specifically, which did not find a significant association between sarcopenia and POPF formation [21].

## 4. Discussion

Many risk factors for POPF after pancreatic surgery are known. In this systematic review, we investigated the impact of sarcopenia on the occurrence of POPF.

The role of sarcopenia in POPF formation after pancreatic surgery, and following pancreatoduodenectomy and distal pancreatectomy specifically, is still controversial in the literature. A better definition of its role could lead to strategies to reduce complications associated with POPF.

The results showed no clear differences in the incidence of POPF between sarcopenic and non-sarcopenic patients undergoing pancreatic surgery. Six of the twenty-one studies observed a statistically significant effect of sarcopenia, but the data showed an unclear picture of its role in POPF formation. According to Nishida et al. [8], and Linder et al. [20], sarcopenia contributed to the occurrence of POPF, while Amrani et al. [15], Sui et al. [17], Box et al. [26], and Tsukagoshi et al. [28] reported that sarcopenia was a protective factor for POPF. Centonze et al. [23] showed a significant difference only for grade C POPF.

An important point to consider is the surgical procedure performed. The occurrence of POPF after pancreatoduodenectomy usually has different causing factors than after distal pancreatectomy. 

Seventeen of the twenty-one studies were conducted on patients undergoing pancreatoduodenectomy, but only five studies [8,17,20,26,28] reported a significant difference in POPF between the two groups. In order to better understand these findings, we also conducted a sub-analysis focusing on the grade of POPF among the studies, as reported in Table 6. Three studies showed a significant difference in the occurrence of clinically relevant POPF (CR-POPF).

Only one study included patients undergoing distal pancreatectomy [21]. There is little evidence in the medical literature on this topic.

Sarcopenia is a common condition among patients undergoing pancreatic surgery, but different definitions and cut-offs have been used to define it. In order to better understand its role in this and other fields, a standardisation of its definition is mandatory.

According to a consensus document elaborated by a Special Interest Group within ESPEN in 2010, diagnosis of sarcopenia should be based on the combined presence of low muscle mass (criterion 1) and low gait speed (criterion 2). Criterion 1 is defined as a percentage of muscle mass ≥ 2 standard deviations below the mean measured in young adults of the same sex and ethnic background. Criterion 2 can be considered as a walking speed below 0.8 m/s in the 4 m walking test [29].

In 2010, the European Working Group on Sarcopenia in Older People defined sarcopenia (EWGSOP) as documentation of low muscle mass (criterion 1) plus documentation of either low muscle strength (criterion 2) or low physical performance (criterion 3). According to the group, one of the techniques that can be used to assess muscle mass in research and routine clinical practice is computed tomography (CT) [30].

In 2019, a revised European consensus on the definition and diagnosis of sarcopenia was published. Probable sarcopenia is identified by low muscle strength (criterion 1). Diagnosis is confirmed by additional documentation of low muscle quantity or quality (criterion 2). If criteria 1, 2, and 3 (low physical performance) are all met, sarcopenia is considered severe. Lumbar third vertebra imaging by computed tomography is considered among the techniques that can be used to detect low muscle mass [31].

We studied the role of sarcopenia in the risk of POPF, but other factors such as visceral adiposity and sarcopenic visceral obesity should be considered and could play a role in this field [9,10,12,18,19,20,32].

Preoperative nutritional status and malnutrition should be carefully evaluated, as malnutrition could be responsible for the attenuated healing process of pancreatic anastomosis.

In order to adequately assess the role of sarcopenia, we should also evaluate data on the state of nutrition of patients, parenteral nutrition, and jejunostomy. The trend of a lower POPF rate in sarcopenic patients than in non-sarcopenic patients found in our study could be explained by the perioperative nutritional supplementation in sarcopenic patients, but more studies are needed to clarify these findings.

The evaluation of sarcopenia, and body composition parameters in general, should be considered in the preoperative risk stratification and the clinical decision making for patients undergoing pancreatic surgery [11,12,14,15]. It can be easily examined on routine preoperative CT scans and could be useful, combined with the assessment of perioperative clinical features, to identify high-risk patients and improve perioperative management strategies [13,19].

In our centre, we carefully evaluate the nutritional status of all patients undergoing pancreatic surgery. Weight loss and BMI are assessed routinely. Serum albumin and protein levels are always considered preoperatively. If surgery can be delayed safely, we avoid operating on patients with albuminemia < 2.8 g/dL and proteinemia < 5.5 g/dL. In these cases, nutritional counselling is performed, and nutritional supplements are provided until the aforementioned values are achieved, if possible, before performing surgery. In case of the detection of sarcopenia in the preoperative CT scans, muscle strength and physical performance should be assessed. Perioperative nutritional supplementation should be considered, especially in high-risk patients.

If the role of sarcopenia in POPF formation were to be confirmed, nutrition therapy and perioperative rehabilitation could help to prevent the occurrence of POPF [17].

Preoperative exercise and nutritional support should be considered and implemented to improve the physical status of patients with a high risk of anastomotic leak. Medications to reduce the risk of POPF such as somatostatin analogues could be used for prophylaxis in selected patients.

We acknowledge the limitations of the present review. First, the radiological definition of sarcopenia varied among the studies. The skeletal muscle index (SMI) at the third lumbar vertebra level on preoperative CT was the most common way of assessing sarcopenia, but the cut-offs were different in the included studies, as well as the indications for surgery and types of resection. Different surgical techniques could lead to a difference in the POPF rate. There was heterogeneity between studies. Most studies were retrospective and conducted in a single institution with small sample sizes. Our results should be confirmed in larger prospective studies before final conclusions can be drawn.

## 5. Conclusions

In conclusion, the relationship between sarcopenia and POPF following pancreatic surgery is still unclear. Only some studies observed a significant difference in the incidence of POPF between the sarcopenic and non-sarcopenic patients undergoing pancreatoduodenectomy. Only one study included patients who underwent distal pancreatectomy. Further studies are needed to better understand the impact of sarcopenia on this surgical outcome and clarify if sarcopenia could really have a protective role in the formation of POPF. Future studies should also take into account the POPF severity and the surgical procedure performed, as they can affect the POPF rate.

## Figures and Tables

**Table 1:** Study characteristics, indications, and types of resection.

Study	Year	Study Design	NOS Scale	Indication	Type of Resection
Nishida et al. [8]	2016	Retro	8	PDAC, bile duct tumour, other	PD, SSPPD
Pecorelli et al. [9]	2016	Pro	9	Periampullary neoplasms, PDAC	PPPD
Sandini et al. [10]	2016	Retro	7	Periampullary neoplasms, PDAC, IPMN, pNET, other	PD, PPPD
Van Dijk et al. [11]	2016	Pro	7	Periampullary neoplasms, PDAC, bile duct tumour, other	PD
Okumura et al. [12]	2017	Retro	8	PDAC	PD, DP, TP
Takagi et al. [13]	2017	Retro	6	Periampullary neoplasms, PDAC, IPMN, other	SSPPD
Van Rijssen et al. [14]	2017	Pro	9	Periampullary neoplasms	PD
Amrani et al. [15]	2018	Retro	8	Periampullary neoplasms, PDAC, IPMN, CP, other	PD, DP, TP
Fukuda [16]	2018	Pro	6	T1D	PTx
Sui et al. [17]	2018	Retro	7	Periampullary neoplasms, PDAC, IPMN, pNET, other	PPPD
Yamane et al. [18]	2018	Retro	7	Periampullary neoplasms, PDAC, IPMN	PD
Jang et al. [19]	2019	Retro	8	Periampullary neoplasms, PDAC, IPMN, pNET, other	PD
Linder et al. [20]	2019	Retro	7	Periampullary neoplasms, PDAC, other	PD, PPPD
Vanbrugghe et al. [21]	2019	Retro	8	PDAC, IPMN, pNET, CP, other	DP, SPDP
Abe et al. [22]	2020	Retro	7	Periampullary neoplasms, PDAC, bile duct tumour, CP, other	PD
Centonze et al. [23]	2020	Retro	8	Periampullary neoplasms, PDAC, IPMN, pNET, CP, other	PD
Roh et al. [24]	2020	Retro	7	Periampullary neoplasms, PDAC, bile duct tumour, IPMN, other	PD
Ryu et al. [25]	2020	Retro	9	PDAC, other	PD, PPPD
Box et al. [26]	2021	Retro	8	Periampullary neoplasms, PDAC, IPMN, pNET, other	PD
Tanaka et al. [27]	2021	Retro	8	Periampullary neoplasms, PDAC, IPMN, pNET, other	PD, DP, MP
Tsukagoshi et al. [28]	2021	Retro	7	Periampullary neoplasms, PDAC, IPMN, pNET, other	PD, SSPPD

NOS: Newcastle–Ottawa Scale; PDAC: pancreatic ductal adenocarcinoma; IPMN: intraductal papillary mucinous neoplasm; pNET: pancreatic neuroendocrine tumour; CP: chronic pancreatitis; T1D: type 1 diabetes mellitus with refractory hypoglycaemia; PD: pancreatoduodenectomy; PPPD: pylorus-preserving pancreatoduodenectomy; SSPPD: subtotal stomach-preserving pancreatoduodenectomy; DP: distal pancreatectomy; TP: total pancreatectomy; SPDP: spleen-preserving distal pancreatectomy; MP: middle pancreatectomy; PTx: pancreas transplantation.

**Table 2:** Sarcopenia measures and cut-offs for each study.

Study	Modality	Level	Measure	Cut-Off
Nishida et al. [8]	CT	L3	SMI (cm^2^/m^2^)	M < 43 (BMI < 25), M < 53 (BMI > 25), F < 41
Pecorelli et al. [9]	CT	L3	TAMAI (cm^2^/m^2^)	M < 52.4, F < 38.5
Sandini et al. [10]	CT	L3	TAMAI (cm^2^/m^2^)	M < 43 (BMI < 25), M < 53 (BMI > 25), F < 41
Van Dijk et al. [11]	CT	L3	Muscle radiation attenuation (HU)	M < 33.9, F < 30.9
Okumura et al. [12]	CT	L3	SMI (cm^2^/m^2^)	M < 47.1, F < 36.6
Takagi et al. [13]	CT	L3	SBI (cm^2^/m^2^)	M < 68.5, F < 52.5
Van Rijssen et al. [14]	CT	L3	SMI (cm^2^/m^2^)	M < 53.5, F < 46.4
Amrani et al. [15]	CT	L3	SMI (cm^2^/m^2^)	M < 52.4, F < 38.5
Fukuda [16]	CT	Umbilicus	PMI (mm^2^/cm^2^) IMAC	M < 303.7, F < 269.4 M > −0.388, F > −0.169
Sui et al. [17]	CT	L3	SMI (cm^2^/m^2^)	M < 40.5, F < 33.5
Yamane et al. [18]	CT	L3	SMI (cm ^2^/m^2^)	M < 43 (BMI < 25), M <53 (BMI > 25), F < 41
Jang et al. [19]	CT/MRI	L3	TAMAI (cm^2^/m^2^)	M < 52.4, F < 38.5
Linder et al. [20]	CT	L3	SMI (cm^2^/m^2^)	M < 43 (BMI < 25), M < 53 (BMI > 25), F < 41
Vanbrugghe et al. [21]	CT	L3	SMI (cm^2^/m^2^)	M < 52.4, F < 38.9
Abe et al. [22]	CT	L3	SMI (cm^2^/m^2^)	M < 43 (BMI < 25), M < 53 (BMI > 25), F < 41
Centonze et al. [23]	CT	L3	HUAC of the psoas muscles (HU)	M < 16.37, F < 14.21
Roh et al. [24]	CT	L3	SMI (cm^2^/m^2^)	M ≤ 52.4, F ≤ 38.5
Ryu et al. [25]	CT	L3	SMI (cm^2^/m^2^)	M < 50.18, F < 38.63
Box et al. [26]	CT	L3	SMI (cm^2^/m^2^)	M < 43 (BMI < 25), M < 53 (BMI > 25), F < 41
Tanaka et al. [27]	CT	L3	SMI (cm^2^/m^2^)	< 44.2
Tsukagoshi et al. [28]	CT	L3	SMI (cm^2^/m^2^)	M < 42, F < 38

SARC: sarcopenia; NSARC: no sarcopenia; CT: computed tomography; MRI: magnetic resonance imaging; L3: the 3rd lumbar vertebra; SMI: skeletal muscle index; TAMAI: total abdominal muscle area index; MRA: muscle radiation attenuation; SBI: skeletal muscle area divided by the body surface area; PMI: psoas muscle mass index; IMAC: intramuscular adipose tissue content; HUAC: Hounsfield unit average calculation; HU: Hounsfield unit; BMI: body mass index.

**Table 3:** Sample size, incidence of sarcopenia and POPF, and comparison between sarcopenic and non-sarcopenic groups.

Study	Patients (n)	SARC (n)	POPF in SARC (n)	NSARC (n)	POPF in NSARC (n)
Nishida et al. [8]	266	132	29	134	14
Pecorelli et al. [9]	202	132	30	70	18
Sandini et al. [10]	124	30	13	94	34
Van Dijk et al. [11]	186	62	10	124	16
Okumura et al. [12]	301	120	10	181	15
Takagi et al. [13]	219	55	20	164	52
Van Rijssen et al. [14]	166	130	38	36	9
Amrani et al. [15]	107	50	18	57	35
Fukuda [16]	41	11	2	30	1
Sui et al. [17]	354	87	17	267	84
Yamane et al. [18]	99	40	8	59	22
Jang et al. [19]	284	191	34	93	18
Linder et al. [20]	139	60	22	79	4
Vanbrugghe et al. [21]	208	156	45	52	20
Abe et al. [22]	136	53	13	83	29
Centonze et al. [23]	110	36	18	74	35
Roh et al. [24]	107	60	12	47	7
Ryu et al. [25]	548	252	15	296	23
Box et al. [26]	220	125	18	95	35
Tanaka et al. [27]	150	74	18	76	12
Tsukagoshi et al. [28]	101	65	9	36	12

SARC: sarcopenia; NSARC: no sarcopenia; POPF: postoperative pancreatic fistula.

**Table 4:** Patients undergoing pancreatic surgery.

	POPF (n)	No POPF (n)	Tot
Sarcopenia (n)	399	1522	1921
No Sarcopenia (n)	495	1652	2147
Tot	894	3174	4068

POPF: postoperative pancreatic fistula.

**Table 5:** Patients undergoing pancreatoduodenectomy.

	POPF (n)	No POPF (n)	Tot
Sarcopenia (n)	306	1204	1510
No Sarcopenia (n)	412	1339	1751
Tot	718	2543	3261

POPF: postoperative pancreatic fistula.

**Table 6:** POPF severity (ISGPF classification) in patients undergoing pancreatoduodenectomy.

Study	POPF Grade	CR-POPF in SARC (n)	CR-POPF in NSARC (n)
Nishida et al. [8]	B and C	29	14
Pecorelli et al. [9]	Any grade	NA	NA
Sandini et al. [10]	Any grade	NA	NA
Van Dijk et al. [11]	Any grade	NA	NA
Takagi et al. [13]	B and C	20	52
Van Rijssen et al. [14]	B and C	38	9
Sui et al. [17]	B and C	17	85
Yamane et al. [18]	B and C	8	22
Jang et al. [19]	B and C	34	18
Linder et al. [20]	B and C	22	4
Abe et al. [22]	B and C	13	24
Centonze et al. [23]	Any grade	14	19
Roh et al. [24]	B and C	12	7
Ryu et al. [25]	B and C	15	23
Box et al. [26]	Any grade	NA	NA
Tanaka et al. [27]	B and C	18	12
Tsukagoshi et al. [28]	B and C	9	12

SARC: sarcopenia; NSARC: no sarcopenia; POPF: postoperative pancreatic fistula; CR-POPF: clinically relevant postoperative pancreatic fistula; NA: not available.

## Data Availability

The data presented in this study are available on request from the corresponding author.

## References

[1] Kamarajah S.K., Bundred J.R., Lin A., Halle-Smith J., Pande R., Sutcliffe R., Harrison E.M., Roberts K.J., PARANOIA Study Group (2021). Systematic review and meta-analysis of factors associated with post-operative pancreatic fistula following pancreatoduodenectomy. ANZ J Surg..

[2] Gianotti L., Besselink M.G., Sandini M., Hackert T., Conlon K., Gerritsen A., Griffin O., Fingerhut A., Probst P., Abu Hilal M. (2018). Nutritional support and therapy in pancreatic surgery: A position paper of the International Study Group on Pancreatic Surgery (ISGPS). Surgery.

[3] Perra T., Porcu A. (2022). State of the Art in Pancreatic Surgery: Some Unanswered Questions. J. Clin. Med..

[4] Ratnayake C.B., Loveday B.P., Shrikhande S.V., Windsor J.A., Pandanaboyana S. (2018). Impact of preoperative sarcopenia on postoperative outcomes following pancreatic resection: A systematic review and meta-analysis. Pancreatology.

[5] Yue Y., Li M., Zhang X., Yu H., Song B. (2020). Prediction of clinically relevant pancreatic fistula after pancreatic surgery using preoperative CT scan: A systematic review and meta-analysis. Pancreatology.

[6] Wells G.A., Shea B., O’Connell D., Peterson J., Welch V., Losos M., Tugwell P. The Newcastle-Ottawa Scale (NOS) for Assessing the Quality of Nonrandomised Studies in Meta-Analyses. http://www.ohri.ca/programs/clinical_epidemiology/oxford.asp.

[7] Bassi C., Marchegiani G., Dervenis C., Sarr M., Abu Hilal M., Adham M., Allen P., Andersson R., Asbun H.J., Besselink M.G. (2017). International Study Group on Pancreatic Surgery (ISGPS). The 2016 update of the International Study Group (ISGPS) definition and grading of postoperative pancreatic fistula: 11 Years After. Surgery.

[8] Nishida Y., Kato Y., Kudo M., Aizawa H., Okubo S., Takahashi D., Nakayama Y., Kitaguchi K., Gotohda N., Takahashi S. (2016). Preoperative Sarcopenia Strongly Influences the Risk of Postoperative Pancreatic Fistula Formation After Pancreaticoduodenectomy. J. Gastrointest. Surg..

[9] Pecorelli N., Carrara G., De Cobelli F., Cristel G., Damascelli A., Balzano G., Beretta L., Braga M. (2016). Effect of sarcopenia and visceral obesity on mortality and pancreatic fistula following pancreatic cancer surgery. Br. J. Surg..

[10] Sandini M., Bernasconi D.P., Fior D., Molinelli M., Ippolito D., Nespoli L., Caccialanza R., Gianotti L. (2016). A high visceral adipose tissue-to-skeletal muscle ratio as a determinant of major complications after pancreatoduodenectomy for cancer. Nutrition.

[11] van Dijk D.P., Bakens M.J., Coolsen M.M., Rensen S.S., van Dam R.M., Bours M.J., Weijenberg M.P., Dejong C.H., Olde Damink S.W. (2017). Low skeletal muscle radiation attenuation and visceral adiposity are associated with overall survival and surgical site infections in patients with pancreatic cancer. J. Cachexia Sarcopenia Muscle.

[12] Okumura S., Kaido T., Hamaguchi Y., Kobayashi A., Shirai H., Yao S., Yagi S., Kamo N., Hatano E., Okajima H. (2017). Visceral Adiposity and Sarcopenic Visceral Obesity are Associated with Poor Prognosis After Resection of Pancreatic Cancer. Ann. Surg. Oncol..

[13] Takagi K., Yoshida R., Yagi T., Umeda Y., Nobuoka D., Kuise T., Fujiwara T. (2017). Radiographic sarcopenia predicts postoperative infectious complications in patients undergoing pancreaticoduodenectomy. BMC Surg..

[14] Van Rijssen L.B., van Huijgevoort N.C., Coelen R.J., Tol J.A., Haverkort E.B., Nio C.Y., Busch O.R., Besselink M.G. (2017). Skeletal Muscle Quality is Associated with Worse Survival After Pancreatoduodenectomy for Periampullary, Nonpancreatic Cancer. Ann. Surg. Oncol..

[15] El Amrani M., Vermersch M., Fulbert M., Prodeau M., Lecolle K., Hebbar M., Ernst O., Pruvot F.R., Truant S. (2018). Impact of sarcopenia on outcomes of patients undergoing pancreatectomy: A retrospective analysis of 107 patients. Medicine.

[16] Fukuda Y., Asaoka T., Eguchi H., Sasaki K., Iwagami Y., Yamada D., Noda T., Kawamoto K., Gotoh K., Kobayashi S. (2018). Clinical Impact of Preoperative Sarcopenia on the Postoperative Outcomes After Pancreas Transplantation. World J. Surg..

[17] Sui K., Okabayshi T., Iwata J., Morita S., Sumiyoshi T., Iiyama T., Shimada Y. (2018). Correlation between the skeletal muscle index and surgical outcomes of pancreaticoduodenectomy. Surg. Today.

[18] Yamane H., Abe T., Amano H., Hanada K., Minami T., Kobayashi T., Fukuda T., Yonehara S., Nakahara M., Ohdan H. (2018). Visceral Adipose Tissue and Skeletal Muscle Index Distribution Predicts Severe Pancreatic Fistula Development After Pancreaticoduodenectomy. Anticancer Res..

[19] Jang M., Park H.W., Huh J., Lee J.H., Jeong Y.K., Nah Y.W., Park J., Kim K.W. (2019). Predictive value of sarcopenia and visceral obesity for postoperative pancreatic fistula after pancreaticoduodenectomy analyzed on clinically acquired CT and MRI. Eur. Radiol..

[20] Linder N., Schaudinn A., Langenhan K., Krenzien F., Hau H.M., Benzing C., Atanasov G., Schmelzle M., Kahn T., Busse H. (2019). Power of computed-tomography-defined sarcopenia for prediction of morbidity after pancreaticoduodenectomy. BMC Med. Imaging.

[21] Vanbrugghe C., Ronot M., Cauchy F., Hobeika C., Dokmak S., Aussilhou B., Ragot E., Gaujoux S., Soubrane O., Lévy P. (2019). Visceral Obesity and Open Passive Drainage Increase the Risk of Pancreatic Fistula Following Distal Pancreatectomy. J Gastrointest. Surg..

[22] Abe T., Amano H., Kobayashi T., Hanada K., Hattori M., Nakahara M., Ohdan H., Noriyuki T. (2020). Preoperative anthropomorphic and nutritious status and fistula risk score for predicting clinically relevant postoperative pancreatic fistula after pancreaticoduodenectomy. BMC Gastroenterol..

[23] Centonze L., Di Sandro S., Lauterio A., De Carlis R., Botta F., Mariani A., Bagnardi V., De Carlis L. (2020). The Impact of Sarcopenia on Postoperative Course following Pancreatoduodenectomy: Single-Center Experience of 110 Consecutive Cases. Dig. Surg..

[24] Roh Y.H., Kang B.K., Song S.Y., Lee C.M., Jung Y.K., Kim M. (2020). Preoperative CT anthropometric measurements and pancreatic pathology increase risk for postoperative pancreatic fistula in patients following pancreaticoduodenectomy. PLoS ONE..

[25] Ryu Y., Shin S.H., Kim J.H., Jeong W.K., Park D.J., Kim N., Heo J.S., Choi D.W., Han I.W. (2020). The effects of sarcopenia and sarcopenic obesity after pancreaticoduodenectomy in patients with pancreatic head cancer. HPB.

[26] Box E.W., Deng L., Morgan D.E., Xie R., Kirklin J.K., Wang T.N., Heslin M.J., Reddy S., Vickers S., Dudeia V. (2021). Preoperative anthropomorphic radiographic measurements can predict postoperative pancreatic fistula formation following pancreatoduodenectomy. Am. J. Surg..

[27] Tanaka K., Yamada S., Sonohara F., Takami H., Hayashi M., Kanda M., Kobayashi D., Tanaka C., Nakayama G., Koike M. (2021). Pancreatic Fat and Body Composition Measurements by Computed Tomography are Associated with Pancreatic Fistula After Pancreatectomy. Ann. Surg. Oncol..

[28] Tsukagoshi M., Harimoto N., Araki K., Kubo N., Watanabe A., Igarashi T., Ishii N., Yamanaka T., Hagiwara K., Hoshino K. (2021). Impact of preoperative nutritional support and rehabilitation therapy in patients undergoing pancreaticoduodenectomy. Int. J. Clin. Oncol..

[29] Muscaritoli M., Anker S.D., Argilés J., Aversa Z., Bauer J.M., Biolo G., Boirie Y., Bosaeus I., Cederholm T., Costelli P. Consensus definition of sarcopenia, cachexia and pre-cachexia: Joint document elaborated by Special Interest Groups (SIG) “cachexia-anorexia in chronic wasting diseases” and “nutrition in geriatrics”. Clin. Nutr..

[30] Cruz-Jentoft A.J., Baeyens J.P., Bauer J.M., Boirie Y., Cederholm T., Landi F., Martin F.C., Michel J.P., Rolland Y., Schneider S.M. (2010). European Working Group on Sarcopenia in Older People. Sarcopenia: European consensus on definition and diagnosis: Report of the European Working Group on Sarcopenia in Older People. Age Ageing.

[31] Cruz-Jentoft A.J., Bahat G., Bauer J., Boirie Y., Bruyère O., Cederholm T., Cooper C., Landi F., Rolland Y., Sayer A.A. (2019). Writing Group for the European Working Group on Sarcopenia in Older People 2 (EWGSOP2), and the Extended Group for EWGSOP2. Sarcopenia: Revised European consensus on definition and diagnosis. Age Ageing.

[32] Pecorelli N., Capretti G., Sandini M., Damascelli A., Cristel G., De Cobelli F., Gianotti L., Zerbi A., Braga M. (2018). Impact of Sarcopenic Obesity on Failure to Rescue from Major Complications Following Pancreaticoduodenectomy for Cancer: Results from a Multicenter Study. Ann. Surg. Oncol..

